# Contrasting Roles of Ang II and ACEA in the Regulation of IL10 and IL1β Gene Expression in Primary SHR Astroglial Cultures

**DOI:** 10.3390/molecules26103012

**Published:** 2021-05-19

**Authors:** Dhanush Haspula, Michelle A. Clark

**Affiliations:** 1 Molecular Signaling Section, Laboratory of Bioorganic Chemistry, National Institute of Diabetes and Digestive and Kidney Diseases, NIH, Bethesda, MD 20892, USA; dhanush.haspulagiridhar@nih.gov; 2 Department of Pharmaceutical Sciences, College of Pharmacy, Nova Southeastern University, Fort Lauderdale, FL 33314, USA

**Keywords:** neuroinflammation, hypertension, cannabinoid, SHR, astrocytes

## Abstract

Angiotensin (Ang) II is well-known to have potent pro-oxidant and pro-inflammatory effects in the brain. Extensive crosstalk between the primary Ang II receptor, Ang type 1 receptor (AT1R), and the cannabinoid type 1 receptor (CB1R) has been demonstrated by various groups in the last decade. Since activation of glial CB1R has been demonstrated to play a key role in the resolution of inflammatory states, we investigated the role of Ang II (100 nM) and/or ACEA (10 nM), a potent CB1R-specific agonist in the regulation of inflammatory markers in astrocytes from spontaneously hypertensive rats (SHR) and Wistar rats. Astrocytes were cultured from brainstems and cerebellums of SHR and Wistar rats and assayed for IL1β and IL10 gene expression and secreted fraction, in treated and non-treated cells, by employing qPCR and ELISA, respectively. mRNA expression of both IL10 and IL1β were significantly elevated in untreated brainstem and cerebellar astrocytes isolated from SHR when compared to Wistar astrocytes. No changes were observed in the secreted fraction. While ACEA-treatment resulted in a significant increase in IL10 gene expression in Wistar brainstem astrocytes (Log2FC ≥ 1, *p* < 0.05), its effect in SHR brainstem astrocytes was diminished. Ang II treatment resulted in a strong inhibitory effect on IL10 gene expression in astrocytes from both brain regions of SHR and Wistar rats (Log2FC ≤ −1, *p* < 0.05), and an increase in IL1β gene expression in brainstem astrocytes from both strains (Log2FC ≥ 1, *p* < 0.05). Co-treatment of Ang II and ACEA resulted in neutralization of Ang II-mediated effect in Wistar brainstem and cerebellar astrocytes, but not SHR astrocytes. Neither Ang II nor ACEA resulted in any significant changes in IL10 or IL1β secreted proteins. These data suggest that Ang II and ACEA have opposing roles in the regulation of inflammatory gene signature in astrocytes isolated from SHR and Wistar rats. This however does not translate into changes in their secreted fractions.

## 1. Introduction

Spontaneously hypertensive rats (SHR) are widely employed for identifying molecular mechanisms of essential hypertension [1,2]. Both in vitro and in vivo studies utilizing this model have uncovered a critical role of the brain renin angiotensin system (RAS) in the pathogenesis of hypertension [3,4]. A hyperactive brain RAS, which includes the primary effector peptide Angiotensin (Ang) II and its receptors Ang type 1 (AT1R) and type 2 (AT2R) receptors, is established as a major factor in the etiology of several cardiovascular diseases [5]. Of particular importance is the AT1R, which is expressed at relatively high levels in the brainstem [6], and its activation results in increased firing frequency in SHR [7].

Multiple reports of a dysregulated inflammatory state in the SHR brainstem can be found in the literature [8,9]. Notably, inflammatory markers such as Interleukin (IL) 6 were altered in the SHR brainstems [8,9]. Additionally, strong evidence of a neuroinflammatory involvement in Ang II-mediated sympathoexcitation has also been reported [10] [11,12]. Interestingly, markers of astrogliosis were also observed in SHR brains, suggesting a role for astroglial inflammation in the pathogenesis of hypertension [13]. In our laboratory, we observed that Ang II could trigger significant surges in IL6 mRNA and protein expression in brainstem astroglial cultures from astroglial cultures obtained from their SHRs and their normotensive control, Wistar rats [14]. Ang receptor blockers have been shown to limit inflammatory responses in brain, suggesting a dependence of neuroinflammatory responses on the central AT1R [15]. More recently, brain RAS and Angiotensin converting enzyme 2 (ACE2) have been implicated in the development of neurological symptoms of COVID-19 infection [16].

Several groups have recently reported extensive crosstalk between the AT1R and the cannabinoid type 1 receptor (CB1R) in several different tissues [17,18,19]. Previously, we observed that Ang II can desensitize and downregulate CB1R in astroglial cultures [20,21]. Intriguingly, astroglial CB1Rs have been demonstrated to diminish inflammatory states in various experimental conditions [22,23,24]. Thus, activation of this receptor may trigger responses that counter-regulate AT1R-mediated potentiation of inflammatory states. This is especially intriguing since leveraging the cannabinoid receptor-mediated anti-inflammatory role has been considered as a therapeutic alternative in several pathological conditions [24], and more recently in COVID-19-related cytokine storm [25]. Hence, we investigated the role of both Ang II and the CB1R-specific agonist, Arachidonyl-2′-chloroethylamide (ACEA), in the regulation of inflammatory states in primary astroglial cultures obtained from SHR and Wistar rats. As a proxy for assessing inflammatory states, we employed both qPCR and ELISA to estimate changes in the gene expression and protein levels, respectively of pro-inflammatory cytokine IL1β, and the anti-inflammatory cytokine IL10 in response to Ang II and ACEA.

## 2. Results and Discussion

### 2.1. Elevated Basal IL10 and IL1β mRNA Expression in Brainstem and Cerebellar Astrocytes from SHR When Compared to Wistar Rats

**Figure 1:**
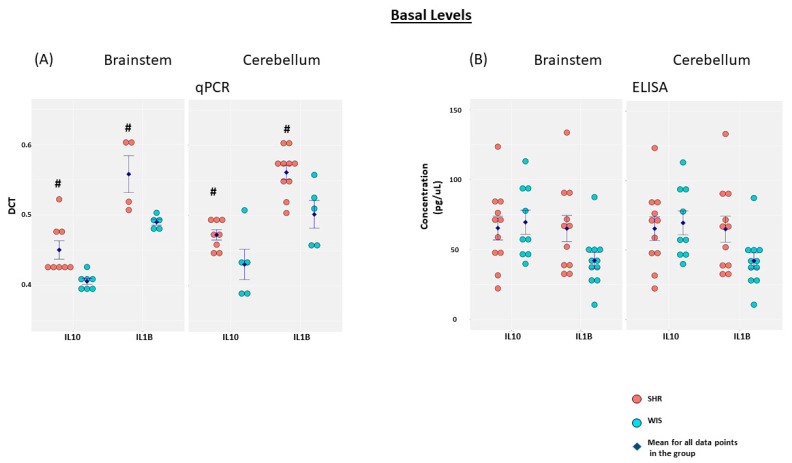
Baseline expression of IL10 and IL1β. Primary brainstem and cerebellar astrocytes, from SHR and Wistar rats, were profiled for IL10 and IL1β mRNA and secreted protein levels by employing (**A**) qPCR and (**B**) ELISA, respectively. The data are represented as a dot plot, with the expression levels shown on the Y-axes, and the different cytokines profiled shown on the X-axes. Different colors are employed for the two rat models. Data shown are the mean ± SEM. # *p* < 0.05 for SHR versus Wistar rat astrocytes. DCT, Differential threshold cycle number; IL, Interleukin.

qPCR and ELISA were employed to determine baseline levels of IL10 and IL1β mRNA and secreted protein, respectively, in both brainstem and cerebellar astrocytes obtained from SHR and Wistar rats (Figure 1). Both IL10 and IL1β mRNA levels were elevated in SHR when compared to the Wistar astrocytes. This elevation in inflammatory gene expression was observed in both brainstem and cerebellar astrocytes. Additionally, IL1β mRNA expression was significantly greater than IL10 mRNA expression in both the brainstem and cerebellar astrocytes obtained from either of the rat models. However, low basal levels of secreted IL1β and IL10 were detected from astroglial cultures, suggesting limited effects on the secretory patterns of the cytokines under investigation from this particular cell type in either hypertensive or normotensive states. Changes in the expression of various cytokines in adult SHR, as opposed to 3-day-old rats employed in our study, have been reported previously [26]. In their study, the levels of IL1β and IL6 were elevated, but IL10 levels were decreased in the cardiovascular centers of the hypothalamus and brainstem of adult SHR when compared to their normotensive controls [26]. While this could be attributable to differences in cell types and experimental design, temporal age-related differences in inflammatory gene signature in SHR brains cannot be ruled out.

### 2.2. ACEA-Mediated Increase in IL10 mRNA Expression Is Significantly Greater in Brainstem Astrocytes of Wistar Rats When Compared to SHR

**Figure 2:**
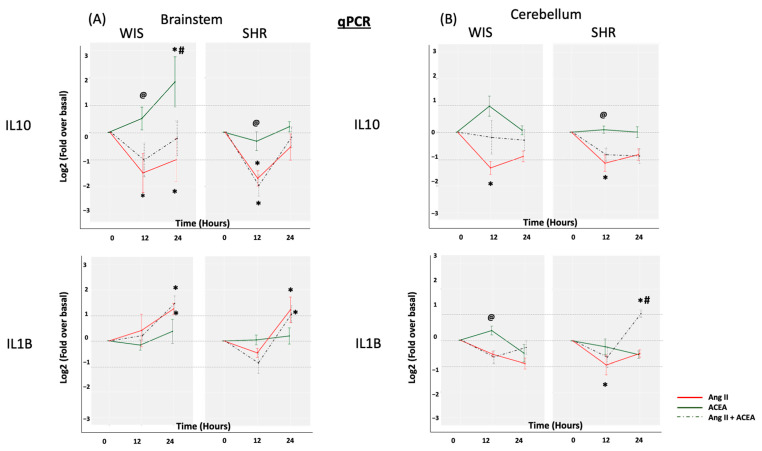
Effect of Ang II and/or ACEA on IL10 and IL1β mRNA expression. Primary (**A**) brainstem and (**B**) cerebellar astrocytes, from SHR and Wistar rats, were profiled for IL10 and IL1β mRNA, in response to Ang II and/or ACEA for 12 and 24 h, by employing qPCR. The data are represented as a line graph, with the change in cytokine expression levels shown on the Y-axes, and the different treatment time points shown on the X-axes. Different colors are employed for the three different treatments. Data are expressed as the mean ±SEM. * Log2FC ≥ 1 or ≤−1 and *p* < 0.05 for treated versus untreated, # *p* <0.05 for SHR versus Wistar rat astrocytes, @ *p* < 0.05 for ACEA versus ACEA + Ang II (co-treatment). Log2FC, Log 2 Fold change for treated versus untreated; Ang II—Angiotensin II.

Both Ang II (100 nM) and ACEA (10 nM) were employed to evaluate the effects of RAS and CB1R activation on IL10 and IL1β mRNA expression in brainstem and cerebellar astrocytes isolated from SHR and Wistar rats (Figure 2). Ang II induced a significant decrease in IL10 mRNA levels in brainstem astrocytes (Log2FC ≤ −1, *p* < 0.05). This effect was similar in both SHR and Wistar rat astrocytes. ACEA treatment however resulted in a greater increase in IL10 mRNA expression in Wistar brainstem astrocytes, but had a diminished effect in the SHR brainstem astrocytes (Log2FC ≥ 1, *p* < 0.05). This is in line with previous studies from our laboratory and others where a reduction in CB1R levels [27,28], and/or hypoactivity of CB1R was reported at the level of MAPKs such as p38 in SHRs [20,21]. P38 has been previously demonstrated to regulate IL10 transcription via the activation of Sp1 transcription factor [29]. In the case of IL1β expression, Ang II triggered a slight increase at 24 h in brainstem astrocytes from both Wistar and SHR. Interestingly, Ang II decreased the levels of IL1β mRNA in cerebellar astrocytes from both Wistar and SHRs. The decrease was statistically significant at 12 h in SHR cerebellar astrocytes. We have previously reported both AT1R and AT2R effects in SHR cerebellar astrocytes [20]. We can only speculate that some of Ang II’s effect in SHR cerebellar astrocytes could be mediated via the AT2R in addition to AT1R. ACEA did not alter IL1β expression in either brainstem or cerebellar astrocytes isolated from either rat strains. Interestingly, the co-treatment of Ang II and ACEA resulted in a trend in both IL10 and IL1β mRNA expression that was mostly similar to Ang II alone in SHR brainstem astrocytes, suggesting that CB1R agonism was insufficient to negate Ang II-mediated changes in inflammatory gene signature under hypertensive conditions. In the case of Wistar brainstem and cerebellar astrocytes, however, co-treatment did not result in a significant decrease in IL10 mRNA expression, suggesting that CB1R agonism may well be able to negate some of AT1R-mediated effects under normotensive conditions. It should also be noted that we did observe an anomalous, yet significant, effect of co-treatment on IL1β mRNA expression in SHR cerebellar astrocytes. At this point, we can only speculate that other crosstalk mechanisms are at play in SHR cerebellar astrocytes.

### 2.3. Neither ACEA nor Ang II Induced Significant Alterations in Secreted IL10 or IL1β from Brainstem and Cerebellar Astrocytes Isolated from Either SHR or Wistar Rats

**Figure 3:**
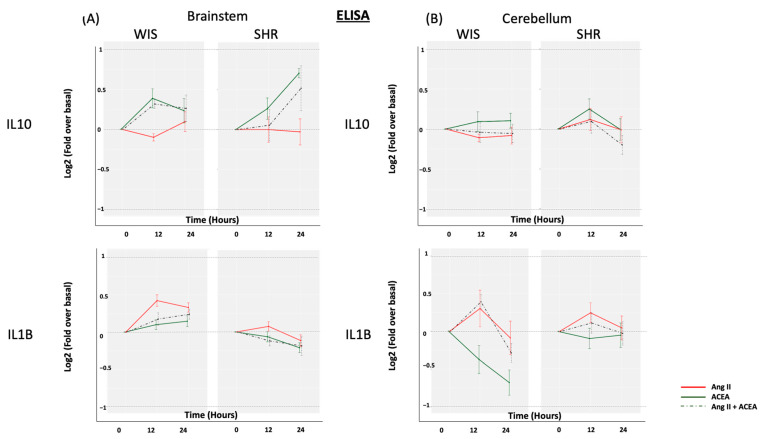
Effect of Ang II and/or ACEA on IL10 and IL1β secreted fraction. Primary (**A**) brainstem and (**B**) cerebellar astrocytes, from SHR and Wistar rats, were profiled for IL10 and IL1β secreted protein levels, in response to Ang II and/or ACEA for 12 and 24 h, by employing ELISA. The data are represented as a line graph, with the change in cytokine expression levels shown on the Y-axes, and the different treatment time points shown on the X-axes. Different colors are employed for the three different treatments. Data are expressed as the mean ±SEM.

Since both Ang II and ACEA induced changes in either/both IL10 and IL1β mRNA expression in SHR astrocytes, we investigated whether this translated to a change in secretory profile from the primary astroglial cultures (Figure 3). Even in the presence of Ang II and/or ACEA, the secreted IL-1β and IL10 fractions from the culture media were below the detection threshold of the ELISA, and required to be concentrated (as per Section 3.4). Hence, an increase of two-fold (Log2FC = 1) was preemptively set as a minimum threshold cut-off. Neither Ang II nor ACEA was able to induce greater than a two-fold change (Log2FC ≥ 1 or ≤ −1) in the secreted fraction of either IL10 or IL1β in the astroglial cell culture media from either rat strains. This suggests that neither Ang II nor ACEA can significantly stimulate the release of IL10 or IL1β from primary astrocytes. Only prolonged treatment (24 h) of ACEA induced a noticeable 1.7-fold increase in the secreted fraction of IL10 in SHR brainstem astrocytes, and a 1.7-fold decrease in IL1β in Wistar cerebellar astrocytes. This small change could well be independent of the changes observed at the level of transcription.

## 3. Materials and Methods

### 3.1. Materials

Ang II was purchased from Bachem (Torrance, CA, USA) and ACEA or Arachidonyl-2’- chloroethylamide from Tocris (Bristol, UK). Sodium deoxycholate (DOC) (89904) was purchased from Thermo Fischer (San Diego, CA, USA). The Taqman primer sets for IL1β (Rn00580432_m1), IL10 (Rn00563409_m1) and beta-actin (Rn00667869_m1) were obtained from Applied Biosystems (Foster City, CA, USA). ELISA kits for IL1β (BMS630) and IL10 (BMS629) were purchased from eBioscience (San Diego, CA, USA). The BCA protein kit was obtained from Pierce Biotechnology (Rockford, IL, USA). All other chemicals were purchased from either VWR International (Suwannee, GA, USA), Fisher Scientific (Waltham, MA, USA) or Sigma (St. Louis, MO, USA).

### 3.2. Isolation and Culture of Primary Astrocytes

Timed pregnant Wistar rats and SHRs, obtained from Charles River Laboratories (Wilmington, MA, USA), were maintained in the ALAAC-accredited animal facility of Nova Southeastern University. Primary astrocyte cultures were prepared using mechanical dissociation as previously described [30]. Briefly, brains from 3-day-old rat pups were isolated, followed by separation of brainstem and cerebellum. These regions are visible and can be clearly differentiated from each other. Astrocyte cultures were then prepared from the pooled brainstem and the pooled cerebellum by physical dissociation. The cells were cultured in DMEM/F12 culture media containing 10% FBS, 10,000 I.U/mL penicillin, 10,000 µg/mL streptomycin and 25 µg/mL amphotericin B at 37 °C in a humidified incubator. On attaining confluency (day 7–9 after plating), the cells were subjected to vigorous shaking overnight which resulted in the detachment of contaminating cells such as microglia and oligodendrocytes. Subsequently, the cell cultures were detached with trypsin/EDTA (0.05% trypsin, 0.53mM EDTA) and replated at a ratio of 1:10 in 100 mm Petri dishes (1 million cells/plate). The astrocyte-enriched cultures were fed once every 3 days until they were about 90% confluent. Before all cell treatments, the cultures were made quiescent by treating for 24–48 h with DMEM/F12 culture media devoid of serum. All subsequent treatments were conducted in serum-free media. The purity of the enriched astrocyte cultures was assessed using Flow cytometry, Western blotting and qPCR as shown previously [20].

### 3.3. Cell Treatments

Astrocyte cultures were treated with 100 nM Ang II and/or 10 nM ACEA for 12 and 24 h on day 12–15 after plating. Astrocytes that were not treated with either Ang II or ACEA were used as the controls.

### 3.4. Total Protein Extraction and Concentration from Conditioned Medium

Immediately following treatments, the conditioned medium was collected and subjected to centrifugation at 1500 rpm for 10 min at 4 °C. The supernatant was collected and stored at −80 °C until further use. Owing to low amounts of secreted IL1β and IL10 in the conditioned medium, the protein was concentrated using the DOC-TCA precipitation method as previously described [31]. The protein was then measured using the BCA assay as per the manufacturer’s instructions. Equal concentrations of proteins samples (1–10 ug) were then employed for ELISA. ELISA for IL1β and IL10 was then performed as per the manufacturer’s protocol.

### 3.5. Total RNA Extraction and mRNA Expression

Total RNA was extracted from astrocytes using the trizol method and subjected to a DNA cleaning step before determining the RNA concentrations using a Bio-Rad SmartSpec^TM^ spectrophotometer. Reverse transcription from total RNA (2 µg) to complementary strand DNA was done using a high-capacity reverse transcription reagent kit (Applied Biosystems). qPCR was performed using the TaqMan Universal master mix, and the TaqMan gene expression primers (Applied Biosystems) for the IL1β and IL10 genes. Samples were analyzed in 96-well plates using the StepOne^TM^plus Real time PCR system from Applied Biosystems. β-actin was employed as the housekeeping gene.

### 3.6. Data Analysis

For the experiments estimating the basal cytokine gene expression, the threshold cycle number (Ct) for every datapoint was normalized to its corresponding reference gene and expressed as DCT. A 2 × 2 mixed ANOVA was employed to estimate any significant differences in the basal expression of IL1β or IL10, between SHR and Wistar rats. This was followed by a Bonferoni T test to determine differences between groups. For experiments determining the effect of Ang II and/or ACEA on cytokines, the change in the gene expression/secreted levels of cytokine of interest is represented as a Log two-fold change (Log2FC). A two-way ANOVA was employed for testing the effect of treatments, both alone and in combination, on IL1β and IL10 in SHR and Wistar rats. A Bonferoni T test was employed to determine significant differences between the various groups. In order to make comparisons between identical time points from different rat models, a two-tailed Student *t*-test was employed. Log2FC ≥ 1 or ≤−1. *p* < 0.05, was set as the threshold for statistical significance to account for both standard error of the datapoint spread, and also the effect size of the various treatments employed. All data are expressed as the mean± SEM for 4 or more biological replicates. Data were analyzed using PRISM (GraphPad). All statistical graphs (dot plots and line graphs) were designed using RStudio.

## 4. Conclusions

An increase in the expression of both the pro- and anti-inflammatory genes, IL1β and IL10, was observed in SHR brainstem and cerebellar astrocytes when compared to their normotensive controls. Incubation of primary astrocytes isolated from normotensive Wistar rats with ACEA resulted in an increase in IL10, and a negligible effect on IL1β mRNA expression. While ACEA treatment resulted in a diminished response in the regulation of IL10 mRNA expression in SHR astrocytes, Ang II exhibited an equally strong reduction in IL10 mRNA in both SHR and Wistar rat astrocytes. Co-treatment resulted in a pattern that was similar to Ang II alone in SHR astrocytes, suggesting that CB1R agonism was insufficient in altering Ang II-mediated effects in SHR. In the case of Wistar astrocytes, co-treatment resulted in negating some of Ang II-mediated effects on IL10 mRNA expression. One major limitation of the study is that neither Ang II, nor ACEA significantly stimulated IL10 or IL1β secretion from primary astrocyte cultures isolated from either rat models. Future studies should also profile other astroglial cytokines involved in various neuropathologies related to cardiovascular disease such as IL8 [32,33]. Our study findings suggests that while both Ang II and ACEA exhibit functionally antagonistic roles in the regulation of IL1β and IL10 mRNA expression in primary astroglial culture, the effects of CB1R agonism may be more pronounced under normotensive, as opposed to hypertensive conditions. Our in vitro data give further credence to the role of both central angiotensin and cannabinoid receptors as potential regulators of inflammatory genes in the brain. Considering that cytokines have a critical role in the pathogenesis of various cardiovascular diseases and COVID-19 related complications, the role of cannabinoids need to be investigated further to potentially limit neuroinflammatory states under in vivo conditions.

## Data Availability

Data are contained within the article.

## Sample Availability

Samples of the compounds are not available from the authors.
